# Stem cells with fused gene expression of cytosine deaminase and interferon-β migrate to human gastric cancer cells and result in synergistic growth inhibition for potential therapeutic use

**DOI:** 10.3892/ijo.2011.1288

**Published:** 2011-12-08

**Authors:** KYOUNG-YOON KIM, BO-RIM YI, HYE-RIM LEE, NAM-HEE KANG, EUI-BAE JEUNG, SEUNG U. KIM, KYUNG-CHUL CHOI

**Affiliations:** 1 Laboratory of Veterinary Biochemistry and Immunology, College of Veterinary Medicine, Chungbuk National University, Cheongju, Chungbuk, Republic of Korea; 2 Division of Neurology, Department of Medicine, University of British Columbia, Vancouver, British Columbia, Canada; 3 Medical Research Institute, Chung-Ang University College of Medicine, Seoul, Republic of Korea

**Keywords:** gastric cancer, therapeutic stem cells, cytosine deaminase, interferon-β

## Abstract

Genetically engineered stem cells (GESTECs) producing suicide enzymes and immunotherapeutic cytokines have therapeutic effects on tumors, and may possibly reduce the side effects of toxic drugs used for treatments. Suicide enzymes can convert non-toxic pro-drugs to toxic metabolites that can reduce tumor growth. Cytosine deaminase (CD) is a suicide enzyme that metabolizes a non-toxic pro-drug, 5-fluorocytosine (5-FC), into the cytotoxic agent, 5-fluorouracil (5-FU). As an immunotherapeutic agent, human interferon-β (IFN-β) has anticancer effects. In this study, we used modified human neural stem cells (HB1.F3) expressing the *Escherichia coli* (*E. coli*) CD gene (HB1.F3.CD) or both the CD and human IFN-β genes (HB1.F3.CD.IFN-β) and evaluated their effectiveness on gastric carcinoma cells (AGS); migration of GESTECs to AGS was analyzed as well as formation of 5-FU and IFN-β. Reverse transcription-polymerase chain reaction (RT-PCR) was used to confirm the expression of CD and IFN-β genes in GESTECs along with confirming the production of chemoattractant molecules such as stem cell factor (SCF), CXCR4, c-Kit, vascular endothelial growth factor (VEGF) and VEGF receptor 2 (VEGFR2). In addition, by co-culturing GESTECs with AGS in the presence of 5-FC, we were able to confirm that cancer growth was inhibited, along with a synergistic effect when the CD and IFN-β genes (HB1.F3.CD.IFN-β) were co-expressed. Indeed a marked anticancer effect was demonstrated when the CD and IFN-β genes were expressed together compared to expression of the CD gene alone (HB1.F3.CD). According to a modified transwell migration assay, the migration of GESTECs toward AGS was confirmed. In conclusion, these data suggest potential application of GESTECs to gastric cancer therapy, due to a remarkable synergistic effect of CD and IFN-β genes in the presence of 5-FC. Additionally, the tumor-selective migration capability *in vitro* suggests that GESTECs are a potential anticancer therapy candidate that may result in minimal side effects compared to the conventional chemotherapy.

## Introduction

Gastric adenocarcinoma is major type of the gastric cancer (1) and in most cases, they are detected in advanced stages. Despite the development of various treatments (e.g., gastrectomy, chemotherapy, radiation therapy) gastric adenocarcinoma ranks second in deaths caused by cancer, although the incidence rate ranks fourth (2). Several drugs are being used in gastric cancer therapy including 5-fluorouracil (5-FU) or its analog capecitabine, BCNU (carmustine) as well as methyl-CCNU (semustine), doxorubicin (Adriamycin), mitomycin C, cisplatin and Taxotere (3,4). Although chemotherapy has been used for a long time, there is no clear standard of care and since gastric cancers are not particularly sensitive to these drugs, chemotherapy is mostly used to reduce the size of the tumor before surgery or used as adjuvant therapy (5). Since the selectivity of the drugs is low, treatments typically include systemic toxicity (3). To reduce these side effects, further studies are needed for safer and efficient treatment for gastric cancer (5–7).

Stem cells have recently become of great interest for researchers with the possibility of clinical use in cancer treatment. While traditional chemotherapy involves administration of manufactured drugs, genetically engineered stem cells (GESTECs) induces cells to produce the therapeutic agent (8,9). This technique enables one to replace damaged genes or insert additional genes with a new function. For example, human neural stem cells (hNSCs) are one of the candidate stem cells showing therapeutic potential and tumor tropism for the treatment of malignant tumors in the human brain including medulloblastomas and gliomas (10–12). This supports the possibility of using hNSCs as a gene carrier to the tumor site as well as a tumor-specific enzyme/pro-drug system with concomitant prodrug administration (13). HB1.F3 is an immortalized hNSC derived from human fetal brain at 15 weeks of gestation by an amphotropic, replication-incompetent retroviral vector v-myc (14,15). Clonal HB1.F3.CD cells were derived from parental HB1.F3 cells transfected with an *Escherichia coli* (*E. coli*) cytosine deaminase (CD) gene (14). Additionally, clonal HB1.F3.CD.IFN-β cells were derived from parental HB1.F3.CD cells and their cells express both *E. coli* CD and human interferon-β (IFN-β) genes (8). This clonally isolated, multi-potent hNSC has the ability to self-renew and to differentiate into cells of neuronal and glial lineages both *in vivo* and *in vitro* (14).

The CD/5-fluorocytosine (5-FC) system is a gene-directed enzyme/pro-drugs therapy (GEPT) (16–20) which converts the non-toxic prodrug 5-FC into the cytotoxic metabolite, 5-FU (21,22). 5-FU inhibits DNA synthesis in cells and results in cytotoxicity (23,24). This CD/5-FC GEPT system has been tested experimentally against several types of tumors including colorectal and prostate cancers (25–27).

In this study, we investigated the synergistic effect of IFN-β with the CD/5-FC GEPT system. The proinflammatory cytokine, IFN-β demonstrated antitumor activity by suppressing angiogenesis, tumor growth and metastasis (28,29). The use of this pro-drug seems to be less toxic compared to using active anticancer drugs, but there is a difficulty in delivering the converting enzymes to the exact tumor site for selective activity. To reduce the side effect of therapeutic drugs and increase their effect, many researchers are focusing on gene-targeting therapy that selectively works on cancer cells (30,31). Therefore, we investigated whether the synergistic effect of the two systems can increase the efficiency of the treatment for gastric cancer.

Its therapeutic capacity in brain tumors as well as its tumor-tropic properties and migratory abilities makes GESTECs a potential candidate for invasive tumors (10–12,32). By delivering genes to selective tumor cells, GESTECs expressing fusion genes (i.e., CD and IFN-β) may have a synergic antitumor effect on gastric cancer cells.

## Materials and methods

### Cell culture

AGS, a human gastric adenocarcinoma cancer cell was originally derived from fragments of a tumor from a patient (Korean Cell Line Bank, Seoul, Korea). The cells were cultured in RPMI (PAA Laboratories GmbH, Linz, Austria) supplemented with 10% (v/v) fetal bovine serum (FBS; Hyclone Laboratories, Inc., Logan, UT, USA), 1% HEPES (Invitrogen Life Technologies, Carlsbad, CA, USA), 1% penicillin/streptomycin (Cellgro Mediatech, Inc., Manassan, VA, USA) and 0.1% antimycoplasmal plasmocin (Invivogen, San Diego, CA, USA) at 37°C in a humidified atmosphere of 5% CO_2_-95% air. HB1.F3, HB1.F3.CD, HB1.F3.CD.IFN-β (Chungang Universuty, Seoul, Korea) and the bovine fibroblast (Bovine FB) cells (Chungbuk National University, Cheongju, Korea) were cultured in DMEM (Hyclone Laboratories, Inc.) supplemented with 10% FBS, 1% penicillin G and streptomycin, 1% HEPES and 0.1% plasmocin at 37°C in a humidified atmosphere of 5% CO_2_-95% air. Cells were trypsinized with 0.05% trypsin/0.02% EDTA (PAA Laboratories) in Mg^2+^/Ca^2+^-free HBSS.

### Reverse-transcription polymerase chain reaction (RT-PCR)

According to recent findings, the tumor tropism of the hNSCs are mediated by several chemoattractants and interaction with their specific receptors including stem cell factor (SCF)/c-Kit (33), stromal cell-derived factor 1 (SDF-1)/CXC chemokine receptor 4 (CXCR4) (34) and vascular endothelial growth factor (VEGF)/VEGF receptors VEGFR1 and VEGFR2 (32). The presence of these chemoattractants and related receptors in AGS were detected by RT-PCR.

Extraction of RNA was performed using the TRIzol reagent (Invitrogen Life Technologies). Using random primers, single-stranded cDNA was synthesized from 1 μg of total RNA by M-MLV RT (iNtRON Biotechnology, Sungnam, Kyeonggido, Korea). The prepared cDNA from this procedure was used in the following PCR reactions performed with 0.2 μmol/l of each sense and antisense primers, 2.5 units of Taq polymerase (iNtRON Biotechnology), 0.2 mmol/l deoxynucleotide mix (iNtRON Biotechnology) and 10X PCR buffer (iNtRON Biotechnology). PCR for these chemoattractant factors (ligands and receptors) and glyceraldehyde-3-phosphate dehydrogenase (GAPDH) as a positive control was carried out for 30 cycles using PTC-100 (MJ Research, Inc., Waltham, MA, USA). PCR cycles were composed of a denaturation reaction at 95°C for 30 sec, annealing reaction at 58°C for 30 sec and extension reaction at 72°C for 30 sec. The results were analyzed on a 1.5% agarose gel containing ethidium bromide (EtBr). The sense and antisense primers and the predicted sizes of the RT-PCR reaction products are shown in Table I.

### Cell growth assay

To investigate the effect of 5-FC and 5-FU in gastric adenocarcinoma cells (4,000 cells/well), AGS were seeded in 96-well plates and cultured in 0.1 ml medium with 5% FBS. After a 24-h pre-incubation, HB1.F3, HB1.F3.CD, and HB1.F3.CD.IFN-β cells were added to the cultures in medium containing 5% FBS and incubated for 24 h before treatment with 5-FC or 5-FU. On the day of treatment, 5-FC and 5-FU (Sigma-Aldrich Corp., St. Louis, MO, USA) were serially diluted with phosphate-buffered saline (PBS; final concentration 100, 200, 300, 400 and 500 μg/ml) and the cells were treated for 4 days. An MTT [3-(4,5-dimethyl-thiazol-2-yl)-2,5-diphenyltetrazolium bromide] assay was performed to measure cell viability on Day 7. MTT solution (10 μl of stock at 5 mg/ml) was added to each well in the plates and incubated for 4 h at 37°C. Supernatants were removed and 100 μl of dimethyl sulfoxide (DMSO, 99.0%; Junsei Chemical Co., Ltd., Tokyo, Japan) was added to each well to dissolve the resultant formazan crystals. Optical densities were measured at 540 nm using an ELISA reader (VersaMan, Molecular Devices, CA, USA). An MTT assay was carried out in duplicate.

To investigate the difference of cell growth and the changes in the ratio of cancer cells to GESTECs, AGS (4,000 cells/well) were seeded in 96-well plates and cultured in 0.1 ml medium with 5% FBS. After a 24-h pre-incubation, HB1.F3, HB1.F3.CD or and HB1.F3.CD.IFN-β cells were added to the cultures in medium containing 5% FBS separately at 8.0×10^3^, 1.6×10^4^ and 2.4×10^4^ cells/well and incubated for 24 h before treatment with 5-FC (Sigma-Aldrich Corp.). On the day of treatment, cells were treated with 5-FC (final concentration 500 μg/ml) for 4 days. MTT assay was performed to measure cell viability on Day 7. MTT solution (10 μl) was added to each well in the plates and they were incubated for 4 h at 37°C. Supernatants were removed and 100 μl of DMSO (Junsei Chemical Co., Ltd.) was added to each well to dissolve the resultant formazan crystals. Optical densities were measured at 540 nm using an ELISA reader (VersaMan, Molecular Devices). The MTT assay was carried out in duplicate.

### In vitro migration assay

To investigate whether GESTECs are capable of migrating to gastric cancer cells, AGS and bovine FB (1×10^5^ cells/well) were plated in 24-well plates and incubated in RPMI and DMEM contained 10% FBS for 6 h at 37°C, respectively. The cells were then incubated with new serum-free media and incubated for 24 h. Transwell plates (8 μm; BD Biosciences, Franklin Lakes, NJ, USA) coated with fibronectin (250 μg/ml; Sigma-Aldrich Corp.) were placed in the 24-well plates and incubated overnight. Using a general protocol, 2 μM of chloromethylbenzamido-1,1′-dioctadecyl-3,3,3′-tetramethyl-indocarbocyanine perchlorate (CM-DiI; Invitrogen Life Technologies) was used to label the HB1.F3, HB1.F3.CD or HB1.F3.CD.IFN-β cells (1×10^5^ cells/well) that were plated in the upper chambers of the transwell plates and cultured in serum-free medium for 24 h at 37°C. The next day, AGS and bovine FB were stained by addition of a 200-ng/ml 4′,6-diamidino-2-phenylindole solution (DAPI; Invitrogen, Lift Technologies) and the plate was incubated for 10 min at 37°C. Each well was washed with PBS and the upper side of the transwell membrane was then scraped to remove cells that had not migrated into the membrane. Cells stained with CM-DiI and DAPI were examined by fluorescence microscopy (IX71 Inverted Microscope, Olympus, Japan).

### Statistical analysis

The results of all cell growth assay experiments are presented as means ± SD. One-way ANOVA was performed and a P<0.05 was considered statistically significant.

## Results

### Confirmation of CD and IFN-β gene expression in GESTECs

**Figure 1:**
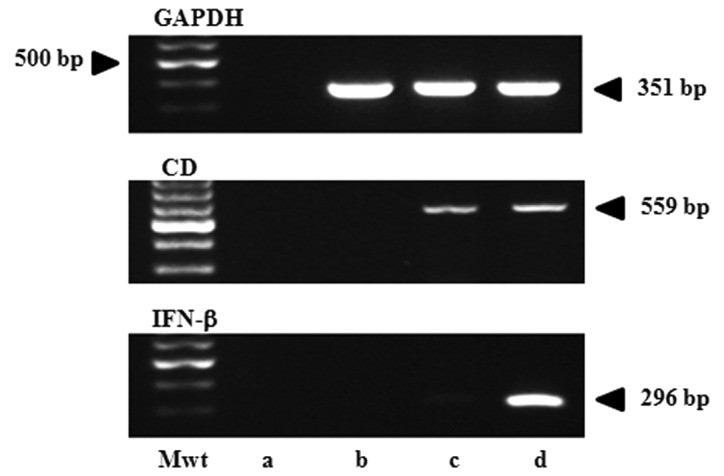
Expression of CD and IFN-β genes in GESTECs. Expected products of *E. coli* CD or human IFN-β genes in HB1.F3.CD and HB1.F3.CD.IFN-β are shown at 559 and 296 bp, respectively. The cDNAs were synthesized from the mRNAs of HB1.F3, HB1.F3.CD and HB1.F3.CD.IFN-β by RT and amplified by PCR. Then, these PCR products were confirmed by agarose gel electrophoresis. GAPDH was used as a control. Mwt, molecular weight marker; a, negative control without template; b, HB1.F3; c, HB1.F3.CD; d; HB1.F3.CD.IFN-β.

The expression of CD and IFN-β genes in HB1.F3, HB1.F3.CD and HB1.F3.CD.IFN-β cells were confirmed by RT-PCR. mRNA of the CD gene (559 bp) was confirmed in both HB1.F3.CD and HB1.F3.CD.IFN-β cells demonstrating CD gene expression in HB1.F3.CD and HB1.F3.CD.IFN-β cells (Fig. 1). In addition, the IFN-β gene (296 bp) was expressed in HB1.F3.CD.IFN-β cells, but not in HB1.F3 and HB1.F3.CD cells (Fig. 1). GAPDH was used as positive control and found based on the presence of its 351 bp cDNA. Results of RT-PCR were confirmed by 1.5% agarose gel electrophoresis.

### In vitro cell migration assay

**Figure 2:**
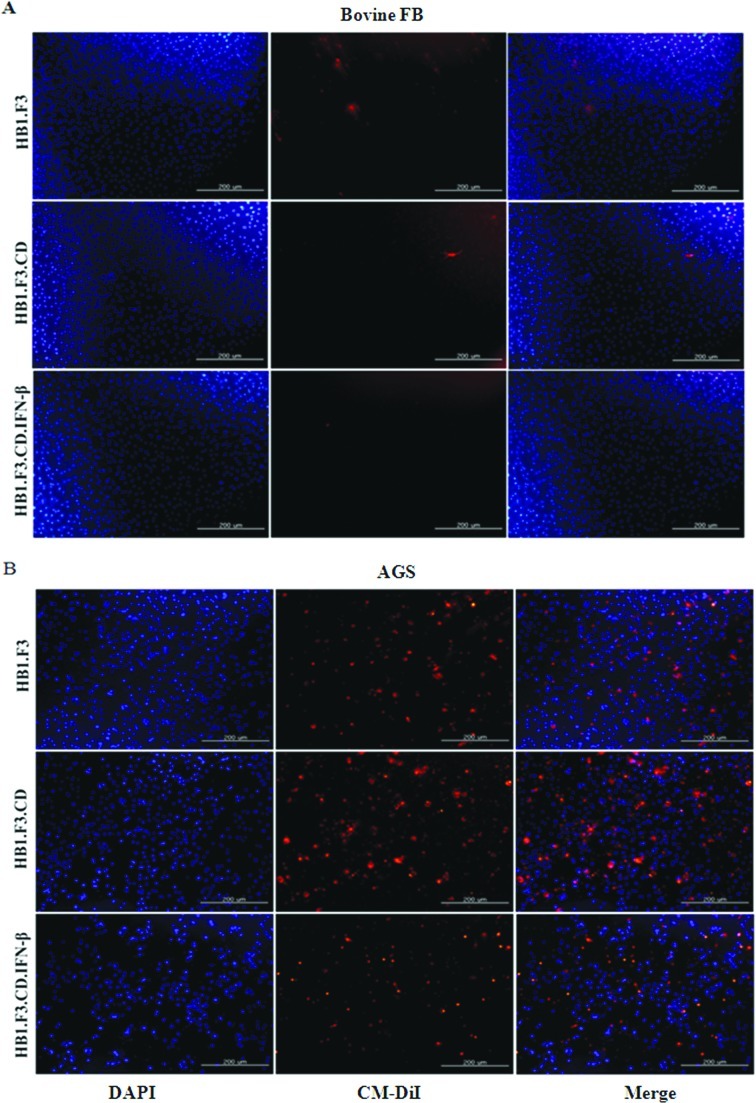
*In vitro* migration of GESTECs toward gastric cancer cells. The migratory capacity was assessed using a modified transwell migration assay. Twenty-four-well plates were pre-coated with fibronectin and HB1.F3, HB1.F3.CD or HB1.F3.CD.IFN-β cells were added to the upper chamber of the insert (1.0×10^5^ cells/well, red stained) after placing the transwell chamber above AGS and bovine FB cells. (A) Bovine FB (1.0×10^5^ cells/well, blue stained) seeded in the chamber. (B) AGS seeded in the lower chamber (1.0×10^5^ cells/well, blue stained). The inserts were collected and stained as previously described. The numbers of cells migrating into the membrane were counted using fluorescence microscopy (x100).

To verify the migration capability of GESTECs toward the AGS, a modified transwell migration assay was performed. Using fluorescence microscopy, changes in CM-DiI stained hNSCs, HB1.F3, HB1.F3.CD and HB1.F3.CD.IFN-β cells was performed. Compared with DAPI-stained bovine FB as a control, AGS significantly increased cell migration of the GESTECs (Fig. 2).

### Confirmation of chemoattractant ligands and receptors

**Figure 3:**
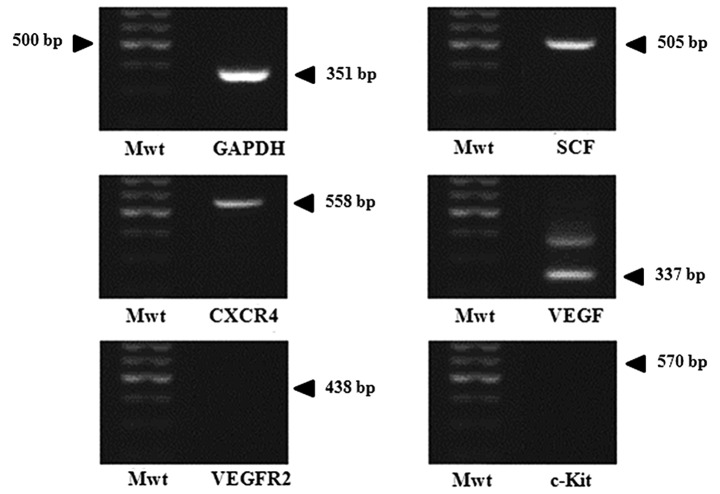
Expression of potential chemoattractant factors involved in tumor tropism and cell growth. The PCR products of GAPDH, SCF, CXCR4, VEGF, VEGFR2 and c-Kit were obtained by RT-PCR as described in Materials and methods. After cDNA synthesis, products were subjected to 1.5% agarose gel electrophoresis. GAPDH was employed as a positive control.

To examine whether gastric cancer cells express chemoattractant factors, RT-PCR for several chemoattractant ligands and their related receptors were done in AGS. Results in Fig. 3 show the expression of SCF, CXCR4 and VEGF genes, but c-Kit and VEGFR2 were not expressed. According to these findings, it can be assumed that AGS produces chemoattractant molecules and related receptors which induce migration of GESTECs.

### Effect of 5-FC/5-FU on gastric cancer cells and GESTECs

**Figure 4:**
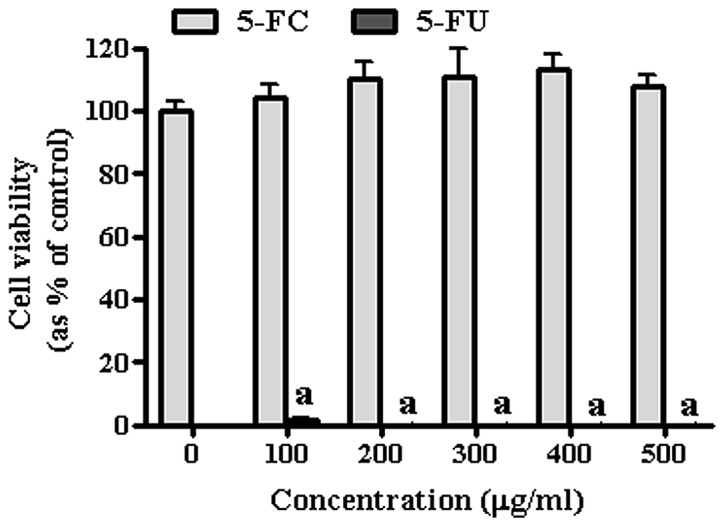
Effect of 5-FC and 5-FU on gastric cancer cell proliferation. Growth of gastric cancer cells were measured following treatments with increasing concentrations of 5-FC or 5-FU (relative fold-change compared to a control). AGS cells (4.0×10^3^ cells/well) were seeded in 96-well plates and were treated with either 5-FC or 5-FU at increasing concentrations (100, 200, 300, 400 and 500 μg/ml). Values represent the means ± SD for three independent experiments. a, P<0.05 vs. 5-FC.

**Figure 5:**
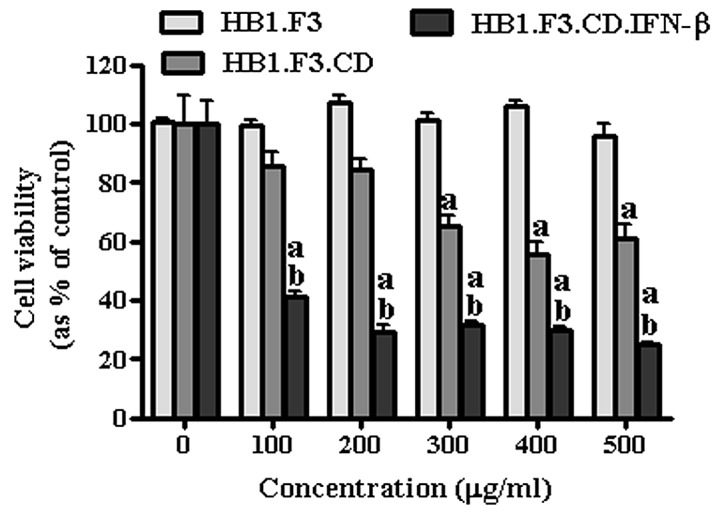
Effect of GESTECs and 5-FC on gastric cancer cell growth. Proliferation of gastric cancer cells were measured following co-culture with GESTECs in the presence of increasing concentrations of 5-FC. AGS cells were seeded in 96-well plates and HB1.F3, HB1.F3.CD or HB1.F3.CD.IFN-β cells (8.0×10^3^ cells/well) were separately co-cultured with AGS, followed by treatment with 5-FC at increasing concentrations (100, 200, 300, 400 and 500 μg/ml). Values represent the means ± SD for three independent experiments. a, P<0.05 vs. HB1.F3; b, P<0.05 vs. HB1.F3.CD.

To confirm the anticancer effect of HB1.F3, HB1.F3.CD and HB1.F3.CD.IFN-β cells, cell viability assay was conducted using a co-culture system and confirmed by MTT assay. Prior to the co-culture experiment with of GESTECs, the effect of the prodrug 5-FC and its active metabolite 5-FU on AGS are shown in Fig. 4. According to these results, 5-FC did not appear to effect the growth of the gastric cancer cells. On the other hand, the growth inhibition effect of 5-FU was significant indicating AGS is highly sensitive to 5-FU, even at low concentration (100 μg/ml) (Fig. 4). To specifically determine the prodrug conversion efficiency of GESTECs, AGS were co-cultured with each stem cell treated by 5-FC at different concentrations (100, 200, 300, 400 and 500 μg/ml) (Fig. 5) and cell viability was measured. HB1.F3 cells, the non-modified control NSC appeared not to inhibit cell growth at any concentration, while HB1.F3.CD cells started to inhibit cancer cell growth with 5-FC treatment reached 300 μg/ml. Impressively, HB1.F3.CD.IFN-β cells showed significant inhibition at the lowest 5-FC concentration (100 μg/ml). In the presence of the GESTECs, treatment of the 5-FC prodrug dose-dependently inhibited cancer cell growth in HB1.F3.CD and HB1.F3.CD.IFN-β cells.

**Figure 6:**
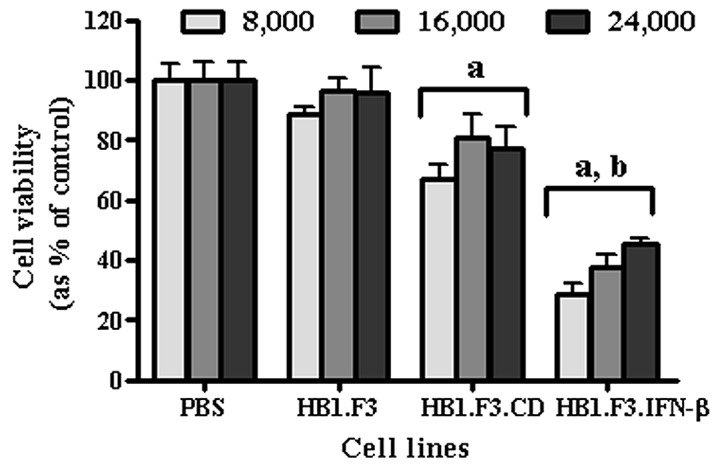
Effect of GESTEC cell numbers on gastric cancer cell proliferation. Proliferative levels of gastric cancer cells were examined following co-culture with increasing numbers of GESTECs in the presence of 5-FC. AGS cells (4.0×10^3^ cells/well) were seeded in 96-well plates, and HB1.F3, HB1.F3.CD and HB1.F3.CD.IFN-β cells were co-cultured with increasing cell numbers (8.0×10^3^, 1.6×10^4^ and 2.4×10^4^ cells/well). Values represent the means ± SD for three independent experiments. a, P<0.05 vs. HB1.F3; b, P<0.05 vs. HB1.F3.CD.

Furthermore, to verify whether the number of the stem cells affected the intensity of anticancer effect in gastric cancer cells, AGS (4.0×10^3^ cells/well) were treated with 5-FC after co-culturing with different amounts of HB1.F3, HB1.F3.CD and HB1.F3.CD.IFN-β cells (8.0×10^3^, 1.6×10^4^ and 2.4×10^4^ cells/well) (Fig. 6). After 5-FC treatment, cell viability was decreased in cells cultured with HB1.F3.CD and HB1.F3.CD.IFN-β cells. Consistent with previous experiments, cancer cell viability was significantly decreased when CD and IFN-β genes were expressed together (HB1.F3.CD.IFN-β).

## Discussion

This study is based on the theory that immortalized GESTECs have potential for gene therapy and cell replacement enabling treatment of neural disease and damage (14,35–40). Among several GESTECs, the NSCs are able to migrate to brain tumor sites and affect tumor growth both *in vitro* and *in vivo* (11,12). In previous studies, using animal models, it was shown that when tumor cells were treated with HB1.F3.CD cells expressing the *E. coli* CD gene and systemic 5-FC administration together, the size of tumor cells were reduced (30,31). At the same time when the tumor was treated only with HB1.F3.CD cells or 5-FC separately, there was no tumor cytotoxicity (12). Additionally, a recent study confirmed that human IFN-β expressing GESTEC, that is HB1.F3.CD.IFN-β cells, showed an anticancer effect compared to HB1.F3 cells (41).

The therapeutic capability of CD gene/5-FC modified GEPT system has been tested in several types of tumors including breast, prostate and colon (20,25,42). The anticancer application of GESTECs is not well investigated in many other cancer cells. Therefore, in this study, we investigated the effect of CD/CD plus IFN-β gene-expressing GESTECs in gastric cancer cells.

First, we tested the direct cytotoxicity of the CD gene/5-FC modified GEPT system with human IFN-β-expressing GESTECs. 5-FU, an inhibitor of thymidylate synthetase (43), has been used to treat cancer for several decades; it causes side effects when administered systemically which include myelosuppression and stomatitis which develop serious complications (3,4). Therefore, to reduce this unwanted effect, the non-toxic prodrug converting *E. coli* CD system has recently received attention from researchers. The CD enzyme, translated from the CD gene converts non-toxic 5-FC into the cytotoxic 5-FU which inhibits cell growth selectively in the site where the gene is expressed (44). In our study, 5-FC-treated HB1.F3.CD cells increased in number when co-cultured with AGS indicating the use of this CD/5-FC GEPT system is possible after the injection of stem cells.

According to earlier reports, it was shown that a small number of CD-transfected cells can induce antitumor effects through a bystander effect (45), thus, we investigated whether the number of the GESTECs induce affected gastric cancer cells differently. When increasing number of the three stem cell lines (8.0×10^3^, 1.6×10^4^ and 2.4×10^4^ cells/well) were cultured with AGS and equally treated with 5-FC at 500 μg/ml, HB1.F3.CD cells expressing the CD gene and HB1.F3.CD.IFN-β cells expressing both the CD and IFN-β fusion genes appeared to show maximum cancer cell growth inhibition starting at a 1:2 ratio of stem cells:AGS, results with higher stem cells number showed similar inhibition effects.

To examine if these gene expressing GESTECs are able to migrate to gastric cancer cells, we performed a modified transwell migration assay. Compared to bovine FB (i.e., control cells), the migration of cells increased in AGS, indicating that gastric cancer cells tend to secrete chemoattractant factors and GESTECs respond to them. In addition, this migrating capability of the parental HB1.F3 cells, was also shown in previous studies using melanoma, glioma, neuroblastoma prostate and breast tumors (11), indicating this cells line possesses a tendency to migrate towards variable types of cancer which can be an advantage for use as antitumor treatment.

Modified migration assay results made it possible to assume that gastric cancer cells might produce chemoattractant factors which induce the migration of HB1.F3.CD and HB1.F3.CD.IFN-β cells to cancer cells, resulting in the delivery of therapeutic genes to the tumor site. Several factors such as SCF, VEGF are known to play a chemoattractive role in tumor cells (10,12,14–27,46–49), but the details in gastric cancer cells are not clearly known. Thus, we assayed for chemoattractant ligands and receptors in AGS and found that SCF, CXCR4 and VEGF genes were expressed. Therefore, these genes may be related in tumor tropism of GESTECs that selectively deliver the suicide enzyme and anticancer cytokine genes to the gastric cancer site. Further study is required to confirm the role of these genes in the mechanism underlying tumor cell recognition and/or tumor tropism by GESTECs.

As explained previously, we studied whether the CD and IFN-β fusion genes can maximize the antitumor effect. Since the mechanism of action of the two genes is different, a possible synergistic effect with the fusion gene was likely. CD acts as a pro-drug-activating enzyme (12) and IFN-β can enhance anti-angiogenic effects and immune responses (31,50). Results from this study showed that HB1.F3.CD.IFN-β cells have significantly powerful antitumor effect compared to HB1.F3.CD cells.

In conclusion, this study showed that the CD gene/5-FC modified GEPT system with the human IFN-β GEPT system resulted in marked growth inhibition in gastric cancer cells. In addition, GESTECs expressing CD or CD with IFN-β genes may selectively migrate toward gastric cancer cells. Therefore, it is possible to consider that GESTECs expressing suicide genes with an application of pro-drugs may have therapeutic potential for treating gastric cancer, and that GESTECs expressing the CD and IFN-β fusion gene has a synergic antitumor effect compared to GESTECs expressing CD alone.

## Figures and Tables

**Table I:** The oligonucleotide sequences of the primers used in this study and the predicted sizes of the PCR products.

mRNA		Oligo-sequences (5′-3′)	Expected size (bp)
CD	Forward	GCGCGAGTCACCGCCAGCCACACCACGGC	559
	Reverse	GTTTGTATTCGATGGCTTCTGGCTGC	
SCF	Forward	ACTTGGATTCTCACTTGCATTT	505
	Reverse	CTTTCTCAGGACTTAATGTTGAAG	
c-Kit	Forward	GCCCACAATAGATTGGTATTT	570
	Reverse	AGCATCTTTACAGCGACAGTC	
CXCR4	Forward	CTCTCCAAAGGAAAGCGCAGGTGGACAT	558
	Reverse	AGACTGTACACTGTAGGTGCTGAAATCA	
IFN-β	Forward	AAAGAAGCAGCAATTTTCAG	296
	Reverse	TTTCTCCAGTTTTTCTTCCA	
VEGF	Forward	AAGCCATCCTGTGTGCCCCTGATG	377
	Reverse	GCTCCTTCCTCCTGCCCGGCTCAC	
VEGFR2	Forward	ACGCTGACATGTACGGTCTAT	438
	Reverse	GCCAAGCTTGTACCATGTGAG	
GAPDH	Forward	ATGTTCGTCATGGGTGTGAACCA	351
	Reverse	TGGCAGGTTTTTCTAGACGGCAG	
